# The continued evolution of team science

**DOI:** 10.1017/cts.2017.4

**Published:** 2017-04-12

**Authors:** Harry P. Selker

**Affiliations:** 1 President, ACTS; 2 Chairman, Clinical Research Forum; 3 Dean, Tufts Clinical and Translational Science Institute

## Introduction

A decade ago, National Institutes of Health (NIH) Director, Elias Zerhouni proposed NIH Clinical and Translational Science Awards (CTSAs) as “homes for clinical and translational science” at their host institutions. This has been an important success of the past decade, but insufficient to accomplish the stated goal to accelerate and improve the quality and impact of research in service of the public’s health.

CTSAs are no longer only primarily directed at creating institutional homes for translational science—that is just a baseline condition. Now CTSAs and other clinical and translational research enterprises are expected to support the capabilities of, and create the conditions for, studies that are relevant and impactful—studies that are responsive to the needs of patients and the public. With crucial nudging from patient-advocacy groups and foundations, and the Patient-Centered Outcomes Research Institute, the bar for broadening inclusion in the planning and execution of research has been raised. We have come to understand that, from inception through completion and dissemination of results, research must have input from intended partners, participants, and beneficiaries. As outlined in the following article, we see this as an evolution of “team science.” With great respect for the hallowed and evolving community-engaged research model, we want to more fully represent the need to authentically include stakeholders, communities, and the broad public as members of the research team: “broadly engaged team science.”

As researchers and leaders of research, members of ACTS and Clinical Research Forum depend on public and individual resources, engagement, and trust. In return, it is incumbent on us to execute research in the public’s interest. This requires application of the best scientific methods, wisely and efficiently—and it also requires engaging the public in new and novel ways. Not only will this generate better and more widely-applicable research, hopefully it will also catalyze a change in how the public regards science, the role of government, the roles of citizenship, and our common mission as members of society.

